# Estradiol as a Targeted, Late-Line Therapy in Metastatic Breast Cancer with Estrogen Receptor Amplification

**DOI:** 10.7759/cureus.1434

**Published:** 2017-07-06

**Authors:** Karthik Kota, Adam Brufsky, Steffi Oesterreich, Adrian Lee

**Affiliations:** 1 Internal Medicine, UPMC Presbyterian; 2 Division of Hematology/Oncology, Magee Women's Hospital of UPMC; 3 Pharmacology and Chemical Biology, University Of Pittsburgh

**Keywords:** estradiol, esr1 amplification, extraordinary responder, metastatic breast cancer, er positive

## Abstract

Estradiol is a major regulator of growth for the subset of breast cancers that express the estrogen receptor (ER, *ESR1*). Strategies to block ER action, via reduction of estradiol or direct inhibition of ER, have shown major success in the prevention and treatment of breast cancer. However, most ER-positive (ER+) metastatic breast cancers (MBC) eventually become resistant to these interventions. Interestingly, high dose estrogen can induce apoptosis in breast cancer cell lines, and high-dose estrogen has been used for over 50 years as therapy for ER+ breast cancer. The mechanism for growth control of MBC by high dose estrogen is unclear. We present a patient with metastatic breast cancer whose tumor was found to have amplification of *ESR1* by tumor genome sequencing. This patient was treated with high dose estradiol and subsequently experienced a sustained partial response, which was predicted by prior experiments with patient-derived xenograft animal models containing breast cancers with ER amplification.

## Introduction

A synthetic estrogen—diethylstilbestrol (DES)—was first used in the 1940s as treatment of metastatic breast cancer (MBC) in postmenopausal females [1]. Breast cancer cell lines that undergo long-term estrogen deprivation can be growth-inhibited by estradiol. High dose estrogen can induce expression of Fas ligand (FasL) in estrogen-deprived breast cancer cell lines [2] and increase Fas receptor (FasR) in tamoxifen-resistant cells [3]; both lead to increased apoptosis via extrinsic pathway activity [4]. High dose estrogen can upregulate mitochondrial pathway proteins (particularly, the ratio of pro-apoptotic B-cell lymphoma-2 (Bcl-2)-associated X (Bax) protein to anti-apoptotic Bcl-2-interacting mediator (Bim) protein) and induce caspase-mediated death in Michigan Cancer Foundation-7 (MCF-7):5C, an estrogen deprived breast cancer cell line. High dose estrogen can also induce p53 localization to tumor mitochondria, where interaction with anti-apoptotic Bcl-2 and Bcl-extra-large (Bcl-XL) frees pro-apoptotic mitochondrial proteins [4]. In all of this work, high dose estrogen led to augmentation in the caspase-mediated apoptosis pathway.

We describe a case of MBC with amplified estrogen receptor (ER, ESR1), detected using next generation sequencing (NGS) and deriving a sustained partial response for many months upon treatment with estrogen. Informed consent statement was obtained for this study.

## Case presentation

**Figure 1:**
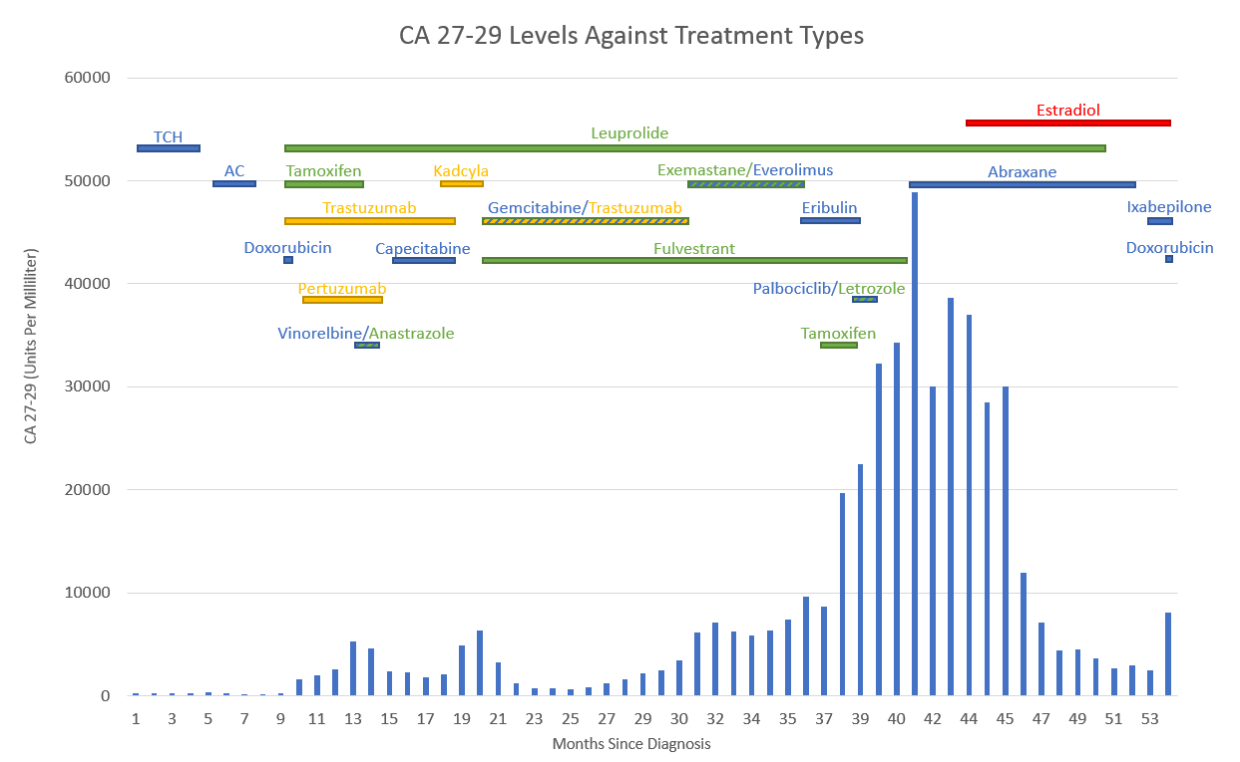
Figure showing the CA 27-29 levels against treatment types New liver metastasis (found to be HER2 negative) identified on computed tomography imaging month 31 (trastuzumab stopped at that time). Estradiol therapy started month 44. The patient dies after month 54

A 36-year-old female who presented after self-palpating a three-centimeter mass in her right breast. She underwent ultrasound-guided core biopsy which revealed invasive ductal carcinoma, nuclear grade three, ER positive (ER+), progesterone receptor (PR) positive, human epidermal growth factor receptor 2 (HER2) equivocal (fluorescence in situ hybridization (FISH) ratio 1.85), and Ki-67 80%. Positron emission tomography (PET)-computed tomography (CT) scan, two weeks later revealed multi-centric disease in the right breast, as well as seven lesions in the liver, the largest being 2.0 x 1.2 cm. The patient began neoadjuvant carboplatin/docetaxel/trastuzumab one month after diagnosis (six cycles was completed four months later) followed by neoadjuvant doxorubicin/cyclophosphamide five months after diagnosis (four cycles was completed two months later). Her liver lesions were noted to respond to this therapy with near complete resolution on PET-CT. The patient then underwent bilateral mastectomy with sentinel lymph node dissection seven months post-diagnosis with near complete resolution of multicentric cancer in her right breast (only ductal carcinoma in situ remained) with one of two sentinel lymph nodes positive with a minute focus (0.1 cm) of adenocarcinoma. Nine months after diagnosis, a laparotomy with planned wedge resection of the liver metastasis was attempted. However, due to extensive small lesions noted on the surface of the liver, resection of a single liver metastasis was performed; the metastasis was well-differentiated adenocarcinoma consistent with breast cancer, strongly ER+. After receiving one dose of intrahepatic doxorubicin (ten months since diagnosis) was managed with multiple subsequent lines of therapy: Tamoxifen/trastuzumab/leuprolide, pertuzumab, trastuzumab/vinorelbine, trastuzumab/anastrozole, trastuzumab/capecitabine, ado-trastuzumab-emtansine, trastuzumab/fulvestrant, and trastuzumab/gemcitabine (Figure 1).

The patients' treatment response was tracked using a combination of computed tomography scanning and serum levels of cancer antigen 27-29 (CA 27-29). Approximately 31 months after diagnosis and after five months of markedly rising serum CA 27-29 levels (all subsequent numbers in units/mL), the patient had a repeat ultrasound-guided liver biopsy which showed adenocarcinoma with estrogen receptors (ER) histology score (H-score) 250, PR H-score 0, HER2 FISH ratio 1.02, and Ki-67 80%; this equivocal FISH result indicated possible partial benefit to trastuzumab. Trastuzumab was discontinued and exemestane and everolimus were begun. Over the next six months, her CA 27-29 slowly rose and her liver lesions progressed on CT scan. Exemestane and everolimus were discontinued and eribulin was started (36 months after diagnosis). As there was not enough tissue from both the patients' liver biopsies for analysis, primary breast tumor from her initial diagnosis was sent (about 37 months later) for next-generation sequencing (NGS) analysis by Foundation Medicine (Cambridge, MA). This testing revealed estrogen receptor alpha gene (ESR1) amplification, a frameshift mutation in GATA-3 protein (GATA3) (p.H434fs) and amplification of zinc finger (ZNF217) [5]. 

**Figure 2:**
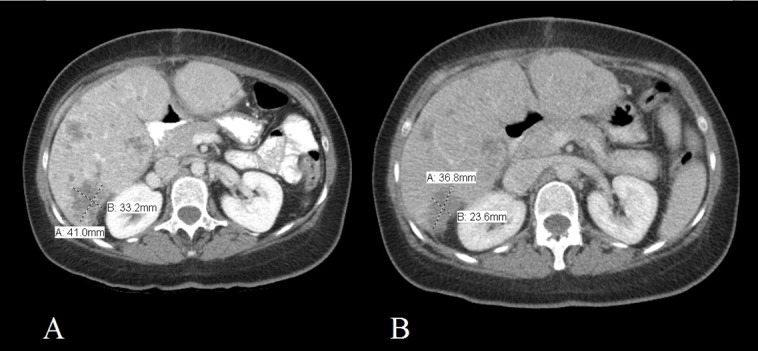
Figure showing the computed tomography of the abdomen with IV contrast Images taken one month before (A) and five months into treatment (B) with estradiol

After further progression on eribulin, palbociclib/letrozole and albumin-bound paclitaxel, 44 months after diagnosis, the patient was started on two milligrams of oral estradiol three times a day. At the start of therapy, the patient's serum CA 27-29 was 28,490, which fell to 12,006 two months later. Four months after starting estradiol, the patient developed scant vaginal spotting and was found to have uterine hyperplasia by pelvic ultrasound and was treated a month later with dilation and curettage. Estradiol was continued. By the time of her CT scan five months into estradiol therapy, the patient had a CA 27-29 of 4517 and noted the decrease in liver metastasis size and number (Figure 2).

After nine months of estradiol therapy, the patients' serum CA 27-29 had dropped to its lowest level (2454; Figure 1), but her baseline bilirubin level rose from 1.7 mg/dL to 3.1 mg/dL. Ixabepilone was added to her estradiol therapy. One month later, estradiol was discontinued. Her malignant ascites with liver failure worsened one week later and 54 months after diagnosis, the patient died of liver failure.

## Discussion

The patient was treated for 303 days with estradiol as her primary anti-cancer agent, with 245 of those days in sustained partial response. Various papers have been published regarding ESR1 mutation and resistance to treatment [6], as well as ESR1 amplification and positive response to endocrine therapy (IE - antiestrogens and aromatase inhibitors) [7]. However, as far as the authors of this work is aware, there are no published reports of using estradiol to treat ESR1 amplification-positive tumors in MBC patients (though preclinical data from a mouse xenograft model [8], discussed below, appears to predict such an outcome).

Estradiol therapy for MBC has been in use for over 50 years, although little is known about its mechanism of action and few randomized trials have been performed. Recently, Ellis, et al. conducted a phase II trial of high-dose (30 mg daily) versus low-dose (six mg) oral estradiol therapy in post-menopausal, ER-positive MBC. Clinical benefit rates (chemical-biological-radiological (CBR), response or stable disease greater than six months) were similar in both high-dose (9/32 (28%, 95% confidence interval (CI): 18%–41%)) and low-dose (10/34 (29% (95% CI: 19%–42%)) arms and no difference in progression-free survival (PFS) or time-to-failure (TTF) was noted. There were, however significantly fewer grade three adverse events (e.g., pleural effusion, pain) in the low-dose arm compared to the high-dose arm [1].

Li, et al. examined ESR1-variant mutations in patient-derived xenografts from hormone resistant MBC. In this study, metastatic tumor from 20 patients was used to make 22 human/mouse xenografts. One xenograft had amplification of ESR1, leading to high levels of ESR1 protein and this patient had marked tumor regression with estradiol [8].

About 30% of patients with MBC develop ESR1 mutations after an extensive period of aromatase inhibitor therapy, requiring a change in endocrine therapy to agents like fulvestrant [6]. The results of the patient NGS showed that she had ESR1 amplification in her primary tumor, which occurs in nearly 20.6% of breast cancers [7]. The strong clinical response suggests that ESR1 amplification is a possible biomarker of response to estradiol.

## Conclusions

Estrogen receptor (ER) is an important predictive biomarker used for treatment in MBC patients. Up to this point, ESR1 amplification has been used as a biomarker for likely benefit of endocrine therapy, while ESR1 mutation has been associated with failure to some forms of antiestrogen therapy and the need for agents such as fulvestrant. Although synthetic and natural estrogens have been used for late-line therapy of ER+ MBC treatment, therapy was mostly based on history of treatments and not in response to expression of a specific marker. The patient's clinical course shows that targeted therapy using NGS to detect ESR1 amplification and treatment with estradiol can lead to a sustained, significant clinic response.
